# Comparative study of visual inspection of the cervix using acetic acid (VIA) and Papanicolaou (Pap) smears for cervical cancer screening

**DOI:** 10.3332/ecancer.2012.262

**Published:** 2012-07-23

**Authors:** SO Albert, OA Oguntayo, MOA Samaila

**Affiliations:** 1 Department of Obstetrics and Gynaecology, ABU Teaching Hospital, Zaria, Nigeria; 2 Department of Pathology, ABU Teaching Hospital, Zaria, Nigeria

## Abstract

This article is a comparative study of two screening methods for pre-invasive lesions of the cervix. The Papanicolaou (Pap) smear, an old and tested screening method, is compared with the findings from visual inspection of the cervix following acetic acid (VIA) wash. VIA is a new screening method being advocated by the World Health Organization as an alternative to Pap smear in low-resource settings.

**Objective:**

The objective of this article is to compare the sensitivity, specificity, positive predictive value, negative predictive value, and accuracy of VIA and Pap smear.

**Methods:**

This was a comparative study carried out in the postnatal clinic of Ahmadu Bello University Teaching Hospital, Zaria. Pap smear samples were taken by the researcher. Samples were fixed in 95% ethyl alcohol and taken to the Pathology Department for interpretation. The cervix was then painted with 3–5% VIA and observed for aceto-white lesions. Suspected areas were biopsied and transported to the Pathology Department for interpretation. Patients with positive Pap smear results were also called back for biopsy. Biopsy served as the reference standard.

**Results:**

There were 351 samples that were suitable for statistical analysis. The sensitivity of VIA was 60%, specificity 94.4%, positive predictive value 50%, negative predictive value 99.4%, and accuracy was 98.6%. Pap smear had a sensitivity of 60%, specificity of 100%, positive predictive value of 100%, negative predictive value of 99.4%, and accuracy of 99.4%.

**Conclusions:**

VIA had a comparable result with Pap smear. It should be incorporated into our national screening programme to complementthe cervical cytology in low-resource settings similar to ours.

## Introduction

Cervical cancer is an important women’s reproductive health problem. It is a preventable disease of significant public health concern especially in developing countries. 83% of more than 493,000 new cases of cervical cancer and 85% of all cervical cancer deaths globally occur in developing countries [1]. Its impact on the lives of women worldwide is indisputable [2].

It is the third most common cancer worldwide and the second most common cancer and leading cause of death from cancer among women in developing countries [2].

Globally, cervical cancer remains an important cause of mortality among young women [2]. In 2005, almost 260,000 women died from cancer of the cervix globally [3]. Nearly 95% of the deaths occur in developing countries [3]. A woman’s lifetime risk of developing and dying from invasive cancer in Nigeria is 2.1% and 1.7%, respectively [4]. From 60 to 80% is seen in advanced stages if diagnosed at all with a low probability of long-term survival. At least 500,000 new cases are identified each year, and more than 90% are in developing countries [3] with rates highest in Central America, sub-Saharan African, and Melanesia [3, 4]. This makes cervical cancer one of the gravest threats to women’s lives [3].

Unlike many cancers, cervical cancer can be prevented. This is because the cervix is easily accessible. This prevention can be achieved using relatively inexpensive technologies to detect abnormal cervical tissue before it progresses to invasive cervical cancer. Most developed countries like the United States saw dramatic reductions in the incidence and death rates from cervical cancer following the implementation of organized screening programmes. Accessibility to treatment, early detection, reduction in parity, and other risk factors have contributed to this decline.

It has been estimated that only about 5% of women in developing countries have been screened for cervical dysplasia in the past five years, compared with about 85% in developed countries [5].

In Nigeria, cervical cancer remains the most common reproductive tract malignancy, and the age adjusted incidence rate is approximately 28.5 per 100,000 [4]. Most cases of cervical cancer are diagnosed predominantly at advanced clinical stages III and IV. Also, as in most other developing countries, no organized screening programme exists.

## Objective

The objective of this study is to compare the sensitivity, specificity, positive predictive value, negative predictive value, and accuracy of visual inspection using acetic acid (VIA) with that of the Pap smear.

## Method

This is a descriptive cross-sectional study.

Subjects were recruited from the postnatal clinic of the Department of Obstetrics and Gynaecology, ABUTH, following the approval of the study protocol by the Ethical Committee of ABUTH.

Clients were counselled on cancer of the cervix, the screening procedures, and objectives of the study, following which consent to take part in the study was sought and obtained.

Questionnaires were filled by the researchers and other doctors who were posted to cover postnatal clinics during the period. This was to obtain information on age, parity, contact bleeding, age at first coitus, number of sexual partners, knowledge, and awareness of cancer of the cervix. Each subject went through naked eye inspection of the cervix and then a Pap smear followed by visual inspection of the cervix after application of 3–5% VIA.

Suspicious or visible lesions on VIA were then biopsied.

Women with abnormal cytology reports were recalled and had biopsies taken, which were sent to the pathologist for analysis.

Confidentiality was maintained by not including their names in the questionnaire in order to elicit the correct response, though information was obtained on how they can be contacted when the results come out so that those who needed to come back for biopsy were reached.

### Exclusion criteria

1. Those who refused to take part in the study.
2. Those with previous abnormal result from previous screening.
3. Those with any visible mass/lesions without application of VIA on the cervix.

The recruited patients were placed in the lithotomy position. The procedure was carried out by the researcher with the assistance of a trained nurse/midwife. An unlubricated sterile Cusco’s bivalve speculum was introduced under good lighting to visualize the cervix. The Ayre’s spatula was used to scrape the transformation zone. This was then smeared on a clean slide and fixed with 95% ethyl alcohol for at least 15 min before transportation to the laboratory for Papanicolaou staining. The Pap smears were interpreted in the Pathology Department of Ahmadu Bello University Teaching Hospital, Zaria, by the Consultant Pathologist.

After taking the cytology specimen, the cervix was painted with a cotton wool soaked in 3–5% VIA. The cervix was examined after 1 min for aceto-white reaction. Suspicious or visible lesions were then biopsied. Tissues were sent in formalin to the laboratory where they were processed. Slides were also read by the pathologist. This served as the reference standard. Patients with abnormal positive Pap smear results were also called back for tissue biopsy. Those with abnormal results were referred for proper follow up and management in the gynaecological clinic.

## Ethical consideration

Participation by all clients in this study was voluntary.

The respondents were assured of confidentiality, and none of the questionnaires bore their names.

Formal approval was obtained from the Ethical Committee of the Ahmadu Bello University Teaching Hospital, Zaria.

Verbal-informed consent was obtained from every subject before being included in the study. Consented clients were made to sign or thumb print.

## Results

Of the total number of clients (359) recruited for this study, eight (2.2%) could not be used because of the inability to get them back for cervical biopsy after abnormal Pap smear results. Of all, 351 clients (97.8%) were suitable for analysis.

Socio-demographic characteristics of the clients show that they were aged between 14 and 45 years with a mean of 28.7 ± 4.2 years (Table 1). 280 clients (79.8%) were multiparous, while 71 (20.2%) were grandmultiparous. All the clients were married; mostly (84.3%) in a monogamous setting (Table 1).

283 clients (63.5%) were Muslims, while the rest were Christians.

**Figure 1:**
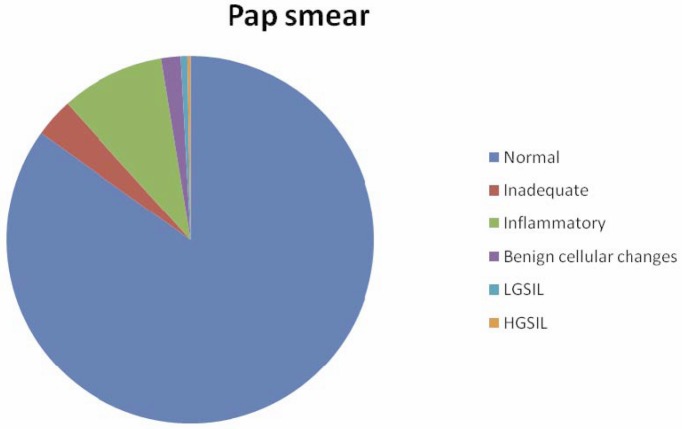
Results of Pap smear.

Pap smear detected 298 (84.9%) as negative (normal), 32 (9.1%) as inflammatory, 6 (1.7%) with benign cellular changes, 2 (0.6%) as LGSIL, 1 (0.3%) as HGSIL, and 12 (3.4%) as inadequate (Figure 1).

VIA was positive in six, Pap smear in three, and cervical biopsy histology in five clients (Tables 2, 3, and 4).

Of the six that were positive to VIA, three were confirmed negative with biopsy histology, while two of those that were negative to VIA but positive to Pap smear were confirmed positive with biopsy histology. These gave a sensitivity of 60%, specificity of 94.4%, positive predictive value of 50%, negative predictive value of 99.4%, and accuracy of 98.6% (Table 5).

Two of those that were negative to Pap smear but positive to VIA were confirmed positive with biopsy histology, while all of those that were positive to Pap smear were confirmed positive by biopsy histology. These gave a sensitivity of 60%, specificity of 100%, positive predictive value of 100%, negative predictive value of 99.4%, and diagnostic accuracy of 99.4% (Table 4).

Those that were missed by VIA were LGSIL.

The prevalence of premalignant lesions of the cervix from this study was 1.4%.

## Discussion

The prevalence of premalignant lesions of the cervix (squamous intraepithelial lesions) of 1.4% noted in this study differs significantly from studies of the same environment and other developing countries. The prevalence noted in the published studies from this environment ranged from 4.8% to 14% [6–9]. A study of Jewish women showed a low prevalence of 0.98% [10]. The prevalence among HIV positive women is even higher, ranging between 10.2% [10] and 34% [11].

The low prevalence noted in this study could be attributable to the fact that the clients used were relatively normal, without any symptoms. They only came to the hospital for follow up after delivery. Those with visible cervical mass/lesions and those with previous abnormal results were excluded from participating in the study.

Many studies have been carried out to compare the sensitivity and specificity of VIA with Pap smear with varying results.

In this study, VIA was noted to have the same sensitivity (60%) and negative predictive value (99.4%) as Pap smear. It has slightly lower specificity (94.4%) and diagnostic accuracy (98.6%) than Pap smear, while its positive predictive value is about half (50%) of that of Pap smear. Over all, VIA seemed to be comparable to Pap smear for screening for pre-invasive lesion of the cervix.

This varied from other studies conducted in this environment [12, 13] but agreed with a study conducted in India [14]. However, in these other studies [15–17], VIA was generally more sensitive but less specific than Pap smear in contrast to this study. A large scale study comparing VIA and Pap smear reports to tissue biopsy reports and HPV typing may help to further evaluate the true state in this environment. This can be done by making use of single or double blinding of the cytopathologists so as to exclude bias.

The results obtained from this study could also be explained by the fact that the VIAs were performed by a single investigator and the Pap smears were also reported by the same person. Thus, this excluded inter-observer variations from both methods.

One of the major reasons for wide variation in results of VIA in many studies is the lack of standardized criteria for a positive result. VIA is also provider dependent. It is thus necessary that before it is used as part of a national screening programme, a uniform reproducible system for categorizing and reporting VIA findings should be put in place. Standard training can then be provided to all health care providers for quality control.

From the data derived from this study, VIA has a comparable sensitivity and specificity with Pap smear. It is also fairly accurate. It can be used as a complementary tool to cytological screening or even when necessary used alone in resource poor countries like Nigeria.

It would reduce the burden of the work on the already burdened cytopathology unit by screening out patients who are VIA negative and disease free. Thus, only patients who are VIA positive would need to undergo further diagnostic test.

It would also reduce the problems of patients that are lost to follow up since the result is immediately available.

The ‘see and treat’ protocol could especially be used in rural areas that are far from cities. Though there is the risk of over treatment, it has to be weighed against the cost benefits of patients lost to follow up who eventually present late diseases.VIA would reduce patients’ anxiety since it would reduce waiting time for the results experienced with cytopathology.

## Figures and Tables

**Table 1:** Socio-demographic data of clients

Characteristics	Frequency	Percentage
Age		
14–19	16	4.6
20–34	276	78.6
35-45	59	16.8
Parity		
1–4	280	79.8
5 and above	71	20.2
Type of marriage		
Monogamy	296	84.3
Polygamy	55	15.7
Sexual partners		
1	273	77.8
2 or more	78	22.2
Coitarche		
19 years and below	146	41.6
20 years and above	205	48.4

**Table 2:** Results of VIA

VIA	Frequency	Percentage
Acetowhite negative	345	98.3
Acetowhite positive	6	1.7
Total	351	100

**Table 3:** Results of biopsy

Biopsy	Frequency	Percentage
Negative	346	98.6
Positive	5 (LSIL 3 & HSIL 2)	1.4
Total	351	100
LSIL: Low-grade squamous intraepithelial lesion, HSIL: High-gradesquamous intraepithelial lesion		

**Table 4:** PAP smear sensitivity and specificity

	Biopsy histology positive	Biopsy histology negative	Total
Pap smear positive	3	0	3
Pap smear negative	2	334	336
Total	5	334	339
Pap smear based on valid result that is minus inadequate smear *Sensitivity*: 3/5 × 100 = 60%, *Specificity*: 334/334 × 100 = 100%, *Positive predictive value*: 3/3 × 100 = 100%, *Negative predictive value*: 334/336 × 100 = 99.4%, *Diagnostic accuracy*: 99.4%			

**Table 5:** Sensitivity and specificity of VIA

	Biopsy histology positive	Biopsy histology negative	Total
VIA positive	3	3	6
VIA negative	2	343	345
Total	5	346	351
*Sensitivity*: 3/5 × 100 = 60%, *Specificity*: 343/346 × 100 = 94.4%, *Positive predictive value*:3/6 × 100 = 50%, *Negative predictive value*: 343/345 × 100 = 99.4%, *Diagnostic accuracy*: 98.6%			

## Conclusion/Recommendations

Mass screening with cervical cytology has reduced the incidence of cervical cancer in developed countries. However, in developing countries like Nigeria, where cervical cancer is the leading cause of death from cancer, there is a dearth of screening programmes. Some of the limitations responsible for this include the lack of resources in terms of trained cytopathologists and cytotechnologists as well as political will.

VIA is of particular interest in developing countries because it is inexpensive, only requires supplies locally obtainable, and can be competently performed by non-physicians with prior training. However, political will is the major factor that will require persistent and focused advocacy to address. This study has shown that VIA is as sensitive as Pap smear and also with comparable specificity and accuracy to Pap smear. VIA is easy, cheap, and treatment can be administered at the same time.

From the findings of this study, it is recommended that VIA can be used to screen for cervical cancer in developing countries where access to Pap smear is lacking. However, Pap smear is still a better method for screening.
